# Low-waste, single-step, sustainable extraction of critical metals from deep-sea polymetallic nodules

**DOI:** 10.1126/sciadv.aea1223

**Published:** 2025-11-21

**Authors:** Ubaid Manzoor, Thomas Lüttke, Dierk Raabe, Isnaldi R. Souza Filho

**Affiliations:** ^1^ Max Planck Institute for Sustainable Materials, 40237 Düsseldorf, Germany.; ^2^ Bundesanstalt für Geowissenschaften und Rohstoffe, 30655 Hannover, Germany.; ^3^ Institut Jean Lamour, CNRS (UMR 7198), Université de Lorraine, F-54000 Nancy, France.

## Abstract

To support the green energy transition, sustainable supplies of critical metals—60 million (metric) tons of copper, 10 million tons of nickel, and 1 million tons of cobalt—annually by 2050 are essential. These metals are currently sourced from declining terrestrial reserves, making deep-sea polymetallic nodules a promising alternative. However, current metal extraction methods are lengthy and energy and carbon intensive, emitting 45, 28, and 4 tons of carbon dioxide equivalent per ton of nickel, cobalt, and copper, respectively. We present a fossil-free hydrogen plasma–based reduction process, powered by green hydrogen and renewable energy, which condenses calcination, smelting, reduction, and refining into a single-step metal extraction, reducing direct carbon dioxide emissions by up to 90% and improving energy efficiency by up to 18%. In addition, we demonstrate selective copper recovery via a heat treatment requiring no acids or reducing agents, offering a more sustainable and cost-effective pathway for critical metal extraction from polymetallic nodules.

## Teaser

A fossil-free hydrogen plasma process enables low-carbon extraction of critical metals from deep-sea polymetallic nodules.

## INTRODUCTION

**Fig. 1.**
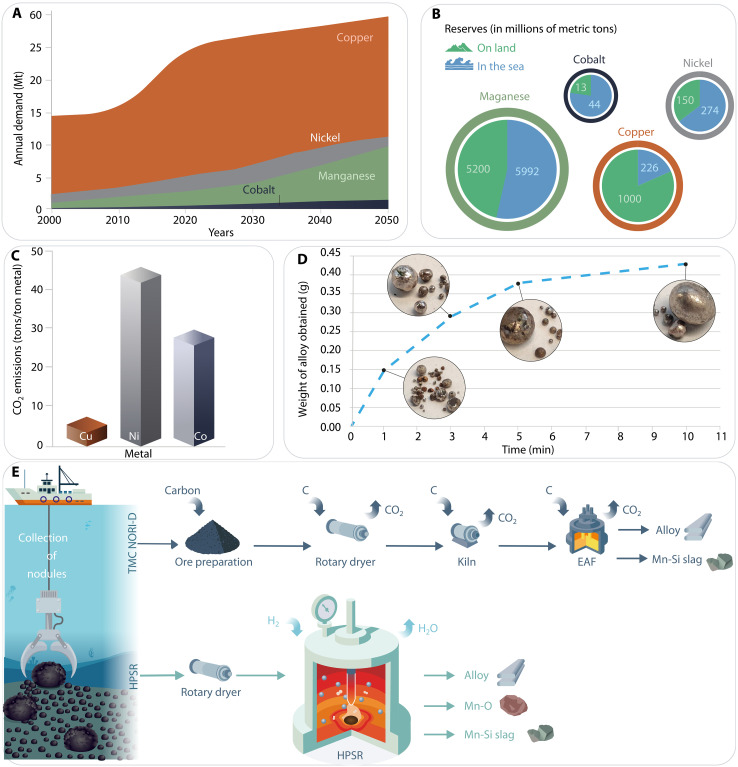
Integrated overview of critical metal production: Market growth, sources, production, processing routes, emissions, and comparison with the sustainable one-step hydrogen plasma route. (**A**) Projected 2050 critical metal market growth, driven by the increasing demand for battery electrode precursor materials required for the electrification of the transport sector (*1*). (**B**) Schematic showing the global distribution of land-based and sea-based critical metal resources (*13*). (**C**) CO_2_ emissions (in tons per ton of metal) from the extraction of metals from terrestrial ores and current carbon-based production routes (*8*–*10*, *14*). (**D**) Weight and photographs of the alloys obtained after hydrogen plasma reduction of nodules at 1, 3, 5, and 10 min. (**E**) Comparative analysis of the current processing route and hydrogen plasma smelting reduction (HPSR) of Mn nodules: The schematic diagram highlights distinct processing steps for the extraction method via TMC NORI-D (The Metals Company Nautilus Ocean Resources Inc., Area D). The one-step HPSR stands out as a single-step process, directly feeding dried ore into a furnace flooded with an Ar-H_2_ mixture. The process produces a refined alloy rich in critical metals (Cu, Ni, and Co), MnO (battery cathode precursor), and Mn-silicate slag (precursor for Mn-Si alloy production). EAF, electric-arc furnace.

As the global community transitions toward a sustainable and low-carbon economy, the demand for critical metals such as nickel (Ni), cobalt (Co), copper (Cu), and manganese (Mn) is increasing. This increase is largely attributed to their indispensable roles in renewable energy technologies, electric vehicle (EV) production, and energy storage systems. Ni, Co, and Mn are vital elements in the production of high-performance lithium-ion batteries, particularly in cathode materials (LiNi*_x_*Mn*_y_*Co*_1–x–y_*O_2_), where they enhance energy density and stability. Cu serves as a crucial conductor in electrical systems, being used in cathodes and anodes of batteries, as well as in the wiring and infrastructure of renewable energy sources such as solar and wind power. Apart from their applications in EVs, Ni and Mn are also used in wind turbines, where Ni enhances the strength and corrosion resistance and Mn improves the durability and wear resistance of critical components, contributing to the overall efficiency and longevity of clean energy technologies. Current projections indicate that global Ni demand is expected to reach ~10 million (metric) tons (Mt) annually by 2050, while Co, Cu, and Mn demands are anticipated to rise to ~1.4, 60, and 19 Mt, respectively (Fig. 1A) (*1*, *2*). This projected increase in demand underscores the urgent requirement for sustainable and reliable sources, value chains, and accessible markets for these essential metals.

Currently, these metals (Ni, Co, and Cu) are extracted from land-based reserves, primarily sourced from Ni laterites ((Mg, Fe, Ni)_3_Si_2_O_5_(OH)_4_ and Ni_3_Si_4_O_10_(OH)_2_.4H_2_O) and sulfide ores (NiS, Ni_2_FeS_4_, (Co,Ni)_3_S_4,_ CuFeS_2_, and Cu_2_S). However, the complexity and diminishing quality of these resources pose challenges to extract these metals in a sustainable and energy-efficient way. The average Ni content (grade) in laterite ores is ~0.5 to 2 wt % Ni, requiring the processing of more than 80 metric tons of ore to extract a single ton of Ni (*1*, *3*). Co, often a by-product of Ni or Cu mining, is typically found in concentrations as low as 0.1 to 0.2 wt %, leading to vast quantities of waste rock and tailings (100 to 200 tons) depending on the ore (Cu or Ni) with which it is associated (*1*). Similarly, Cu mining has seen a decline in ore quality, and historically rich deposits containing more than 2 wt % Cu have been largely exhausted, leaving ores with an average grade of only 0.6 wt %, which results in around 200 tons of waste for every ton of Cu produced (*4*, *5*). These inefficiencies contribute to an alarming statistic: Current mining operations (from land-based ores) of Cu, Ni, and Co produce around 4 to 5 billion (metric) tons (Gt) of waste rock and tailings annually, often laden with toxic heavy metals and acids, posing severe environmental risks (*6*, *7*).

Conventional methods for extracting these metals are energy intensive and contribute to CO_2_ emissions. For instance, processing Ni laterites through rotary kiln–electric-arc furnace (RK-EF) smelting results in ~45 to 48 tons of CO_2eq_ emissions per ton of Ni produced (*8*, *9*). Similarly, the Co extraction methods, depending on the ore in which Co is present, emit around 28.2 tons of CO_2eq_ per ton of Co extracted (*10*). Cu mining also causes environmental harm, with a CO_2_ emission of ~4.1 tons per ton of Cu produced (*11*). This complexity of extraction not only exacerbates the environmental impact but also highlights the need for more efficient and sustainable approaches to metal production (*12*).

In light of these challenges, polymetallic deep-sea nodules, particularly those located in the Clarion-Clapperton zone (CCZ) of the Pacific Ocean, present a promising alternative. These nodules contain high concentrations of critical metals, typically comprising 27% Mn, 1 to 2% Ni, 0.5 to 1% Cu, and 0.1 to 0.2% Co. Recent estimates indicate that the resource base of critical metals in the CCZ may surpass the land-based reserves especially for Ni and Co, presenting an attractive opportunity to address the increasing global demand for these essential materials (Fig. 1A). The CCZ is estimated to contain ~5992 Mt of Mn, 274 Mt of Ni, 226 Mt of Cu, and 44 Mt of Co (Fig. 1B) (*13*). By comparison, currently reported land-based reserves for Ni and Co are lower, estimated at only 150 and 13 Mt, respectively (*13*). These figures for land-based reserves represent only the present economically recoverable reserves, which are subject to change with technological and economic developments. The CCZ figures, in contrast, reflect a potential resource base rather than formally classified reserves. As an illustration of the solid waste generated in the production of 1 billion EVs, two supply scenarios can be compared: land-based ores and polymetallic nodules from the deep sea. According to Paulikas *et al.* (*14*), producing battery metals from land-based ore sources, including overburden, tailings, and processing and refining residues, would yield ~63 Gt of solid waste, whereas using deep-sea polymetallic nodules, including sediment disturbance during collection plus comparable processing/refining residues, would produce only about 9 Gt of waste under the baseline scenario. This is mainly due to the vertical nature of the deposit, i.e., they occur without overburden and contain multiple critical metals (Cu, Ni, and Co) within a single deposit (*12*). In contrast, laterite mining (for Ni and Co) requires extensive land clearance and generates large volumes of overburden, contributing to deforestation, soil erosion, and ecosystem disruption (*12*). These disparities underscore the potential of deep-sea polymetallic nodules as a more sustainable alternative to land-based ores for supplying critical metals to the green technology sector. However, many proposed extraction methods replicate the carbon-intensive and chemically burdensome practices used in terrestrial mining, raising concerns that environmental harm may simply be relocated rather than reduced. For instance, hydrometallurgical techniques such as High-Pressure Acid Leaching (HPAL) and Atmospheric Leaching, which are commonly used for Ni and Co recovery from laterites, have also been explored for seafloor nodules. However, these methods face sustainability challenges beyond carbon emissions. HPAL of laterites, in particular, consumes 30 to 50 kg of sulfuric acid per ton of feed and up to 5 m^3^ of fresh water, generating increasing reagent demand and placing pressure on local water resources (*15*, *16*). In addition, the complexity of these processes, especially when applied to multielement feedstocks such as deep-sea nodules, results in operational inefficiencies and limits flexibility (*15*, *17*). Effluents from such processes require extensive neutralization and pose serious environmental risks if not properly contained. The compositional differences make process parameter values derived from laterite HPAL systems poorly applicable to nodules. Alternative hydrometallurgical processing methods, such as sulfuric acid, hydrochloric acid or chlorination leaching, and ammoniacal leaching, are often combined with reductants such as Fe^2+^, SO_2_, or more recently biogenic organics such as glycerol to enhance the dissolution kinetics of Mn, Ni, Cu, and Co (*18*–*22*). In particular, glycerol-assisted H_2_SO_4_ leaching has been shown to achieve >95% Ni and >98% Cu, Co, and Mn recovery within 1 hour at 80°C, with glycerol oxidation pathways accelerating reductive kinetics (*18*). However, despite these advantages, such approaches still require substantial quantities of sulfuric acid, generate potentially harmful effluents, and exhibit kinetic limitations under certain operating conditions, raising concerns regarding large-scale sustainability (*20*–*22*). These limitations highlight the urgent need for alternative metallurgical pathways that minimize both carbon emissions and chemical waste, enabling more robust sustainable utilization of polymetallic nodules.

Researchers have investigated hydrogen reduction of Mn nodules in the solid state, but the complex mineralogical associations of metals within the Mn-oxide matrix slow down the reduction kinetics (*23*, *24*). These metals are not present as individual oxides but are instead dissolved within the Mn-oxide matrix (*25*), forming complex associations with the matrix having a semicrystalline halite such as crystal structure (*25*–*27*), which makes their reduction very challenging. As a result, complete reduction of metal oxides is not achieved, and additional steps such as magnetic separation and hydrometallurgical processing are required to effectively recover the partially reduced metals. Fossil-based high-temperature smelting of nodules is also practiced (*28*), with the TMC NORI-D [The Metals Company Nautilus Ocean Resources Inc. D (area code)] project commercially processing nodules using the conventional multi-step, carbothermal RK-EF technology route (Fig. 1E) (*29*). In this approach, the nodules are first dried in rotary dryers and then calcined in rotary kilns at ~900°C with carbonaceous material (e.g., coke) and under the flow of natural gas (CH_4_). Coke and natural gas act as heat sources and reducing agents, partially reducing Cu, Ni, Co, and Fe oxides to their metallic states and producing CO_2_ as a by-product. The calcined charge is then transferred to the electric-arc furnace, where additional carbon is added for complete metal oxide reduction, forming an alloy hosting Ni, Cu, and Co, along with an unreduced slag phase rich in Mn_2_SiO_4_. The alloy then undergoes hydrometallurgical refinement to produce battery-grade materials such as Cu cathodes, Ni sulfate, and Co sulfate. The use of solid carbon as both a heat source and a reducing agent leads to high CO_2_ emissions (~5 tons per ton of alloy) and high energy demands due to the endothermic nature of reduction reactions at high temperature involving solid carbon as reducing agent (*29*, *30*). In addition, the final alloy produced contains impurities such as Si, S, and P, necessitating further refinement toward higher purity.

Here, we present hydrogen plasma smelting reduction (HPSR) as a sustainable, energy-efficient, partially electrified and fossil-free alternative for polymetallic nodule processing. HPSR facilitates the breakdown of complex mineralogical structures into simpler ionic components by melting the feedstock, enabling rapid and efficient metal reduction. The exothermic nature of oxygen removal reaction by hydrogen plasma species enhances thermal energy transfer to the reaction interface (*31*), accelerating reduction kinetics and enabling effective single-step slag-metal separation and alloy formation at elevated temperatures. Using hydrogen as a reducing agent, this method produces a refined alloy directly from dried nodules in a single smelting-reduction step (Fig. 1E), eliminating CO_2_ emissions by avoiding carbon-based reductants and preventing sulfur contamination of the alloy typically caused by coke (Fig. 1E). On the basis of preliminary calculations, energy savings of up to 18% can be achieved by bypassing the calcination step, while CO_2_ emissions can be reduced by up to 90% compared to the conventional RK-EF–based TMC NORI-D process, provided the energy and hydrogen are procured from renewable sources (more detailed calculations in the Supplementary Materials section “Energy and CO_2_ emission comparisons”). The metals obtained from HPSR are extracted in the form of an alloy, as shown in Fig. 1D. This figure illustrates the amount of alloy obtained in grams after processing 10 g of the polymetallic nodules over increasing periods of time, ranging from 1 to 10 min.

This work thus provides the scientific foundations and engineering perspectives on the viability of using polymetallic nodules from deep-sea mining as a future key multimillion-ton feedstock source for the sustainable and globally accessible production of some of the most critical metals required for making society and industry more sustainable through electrification. While these nodules contribute to more sustainable, reliable, accessible, tariff-free, and robust supply chains for these critical metals, understanding the full scope, plasmachemical foundations, and environmental impacts and benefits associated with different extraction and processing methods is essential. It is crucial to consider both the promise of deep-sea resources and the unknown ecological consequences of deep-sea mining, ensuring a comprehensive understanding before committing to any large-scale and systematic exploitation. We therefore provide here scientifically grounded insights into the thermodynamics, microstructural evolution, and chemical partitioning involved in the HPSR of polymetallic nodules. The results from this study can also be used as information basis required for future market developments and legislative decision-making regarding the general future of deep-sea mining and utilization of mineral resources from a so far gigantic untapped reservoir without falling into the trap of repeating “old mistakes” because of insufficient scientific scrutiny of the associated downstream refinement and extraction operations. Beyond these fundamental aspects of this work, our study also provides first insights into the formation mechanism of a quintuple “high-entropy–type” alloy using HPSR in a single step from a complex multimetal oxide system, such as that present in polymetallic deep-sea nodules. We thereby thus not only represent a sustainable approach for extracting valuable metals from nodules but also reveal the synthesis of multimetal alloys in a single extraction, processing, and blending step directly from the naturally occurring ore mineral. This is in stark contrast with conventional alloy production, which typically involves the extraction of individual metals from individual and metal-specific ores, followed by the melting and mixing of these metals to achieve the final alloy composition (*32*). Although the resulting multimetal alloy is not designed intentionally but rather forms as a consequence of the polymetallic feedstock chemistry, our study provides thermodynamic and phase transformation insights into high-temperature interactions during smelting, offering a foundation for future alloy design considerations. Through this research, we seek to advance the scientific understanding of alloy production from complex multimetal oxide systems while addressing the sustainability challenges of traditional metallurgical processes.

## RESULTS

### Reductant-free pure Cu formation via in situ oxygen exchange

**Fig. 2.**
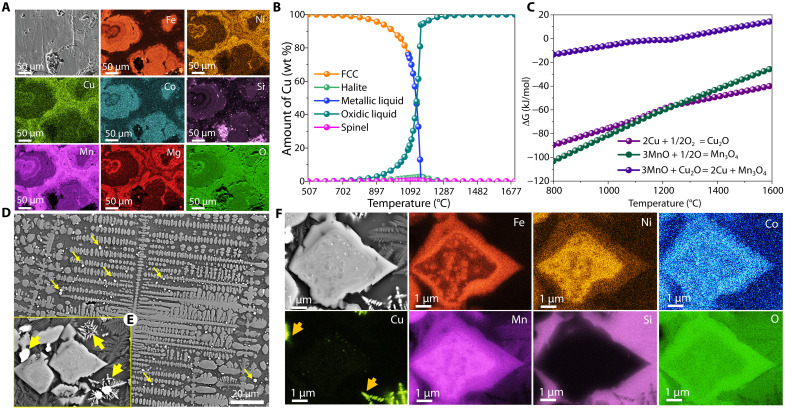
Elemental distribution, thermodynamic analysis, and microstructural evolution during Cu precipitation. (**A**) Elemental distribution determined by SEM-EDX analysis in as-received Mn nodules. (**B**) Thermodynamic calculations illustrating Cu partitioning into different phases upon solidification of the molten sample after melting in Ar. At temperatures above 1100°C, Cu remains dissolved in the oxidic liquid, while below this temperature, it partitions into pure Cu (metallic liquid; blue line) and subsequently into the face-centered cubic (FCC) phase (orange line). (**C**) Gibbs free energy changes for the oxidation of Cu to Cu_2_O (light purple) and MnO to Mn_3_O_4_ (dark green) indicate that these reactions intersect at ~1200°C, implying that MnO can reduce Cu_2_O to metallic Cu below this temperature via the reaction Cu_2_O + 3MnO → 2Cu + Mn_3_O_4_. The corresponding ΔG versus T curve (dark purple) confirms the thermodynamic feasibility of Cu precipitation from the melt (ΔG < 0) below 1200°C. (**D**) Microstructure of a solidified sample reduced in Ar for 2 min, showing Cu solidification within a dark phase, with yellow arrows indicating fine, uniformly distributed Cu precipitates. The magnified view in (**E**) (yellow frame) highlights the morphology of these Cu precipitates (yellow glowing arrows). (**F**) SEM-EDX elemental mapping of the micrograph in (E) reveals that Ni, Co, and Fe partition into the Mn-oxide–rich phase, while Cu precipitates as dendrites in metallic form (orange arrow).

The as-received polymetallic deep-sea nodules were characterized using microwave plasma–atomic emission spectroscopy (MP-AES) and x-ray diffraction (XRD) to assess their chemical composition and phase constitution, as outlined in tables S1 and S2, respectively. MP-AES analysis revealed the presence of 1.2 wt % Ni, 1 wt % Cu, 0.2 wt % Co, and 27.7 wt % Mn, along with other elements. XRD showed predominantly only partially crystalline phases, limiting the identification and precise quantification of phase constituents, a characteristic frequently reported for such raw materials. The identifiable crystalline phases included MnO_2_.0.3H_2_O (~80 wt %), SiO_2_ (~4 wt %), Fe-Mg-Mn-Al hydroxide (~7 wt %), and clay mineral albite Na_0.6_Ca_0.4_Al_1.4_Si_2.6_O_8_ (9 wt %). On the basis of these findings and existing literature (*13*, *25*, *27*), it can be inferred that the critical metals Cu, Ni, and Co are not present in these nodules in the form of discrete binary oxide phases but rather are incorporated into chemically highly mixed complex Mn- and Fe-based oxide matrices. The complex mineralogical association is further corroborated by scanning electron microscopy with energy-dispersive x-ray spectroscopy (SEM-EDX), as illustrated in Fig. 2A. Elemental mapping indicated that Ni and Cu are associated with the prevalent Mn-oxide matrix, while Co predominantly partitioned into the Fe-Mn oxide phases. This intricate intergrowth of mixed metal oxides makes the efficient extraction of Ni, Cu, and Co very challenging and costly using conventional solid-state reduction methods.

**Fig. 3.**
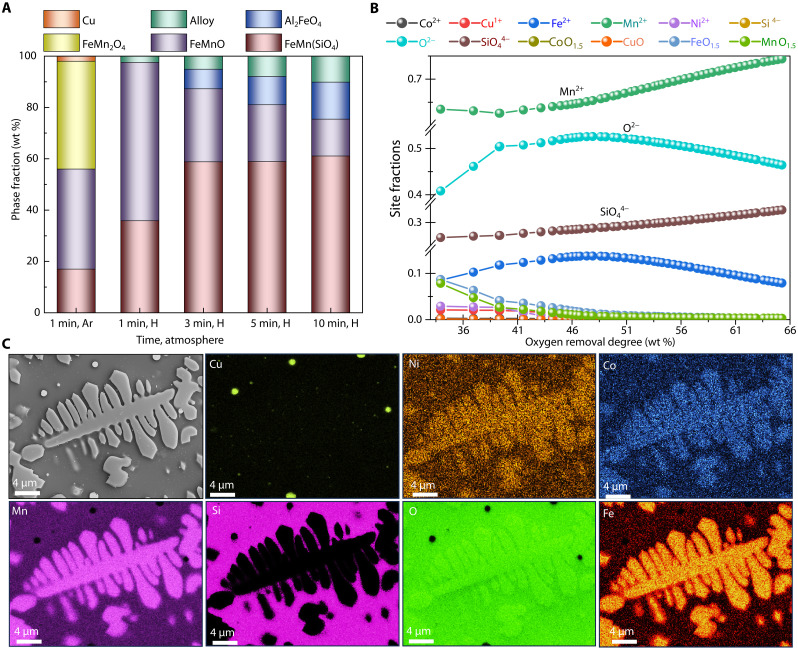
Phase evolution, ionic species transformation, and microstructural development during the melting of Mn nodules in inert and reducing atmospheres. (**A**) Phase evolution within the solidified sample, as determined by Rietveld refinement of XRD patterns (uncertainty: ±1 wt %): The first column shows that melting in an inert Ar atmosphere for 1 min (1 min; Ar) transforms the sample primarily into two Mn-oxide phases: FeMnO (39 wt %) and FeMn_2_O_4_ (42 wt %), FeMnSiO_4_ (17 wt %), and precipitation of pure Cu (2 wt %). Upon exposure to a hydrogen plasma (Ar–2.5% H_2_), alloy formation occurs at the expense of the Mn-oxide phases (FeMn_2_O_4_ and FeMnO), indicating that FeMnO acts as a host for critical elements throughout the reduction process. (**B**) The site fractions of various ionic species within the molten Mn nodules (detailed in Materials and Methods) reveal a transition from a complex Mn nodule into a simpler ionic mixture containing species such as Fe^2+^, Ni^2+^, CuO, Co^2+^, Mn^2+^, SiO_4_^4−^, and O^2−^. Exposure to hydrogen leads to the elimination of free oxygen (O^2−^), which triggers the precipitation of metallic phases. (**C**) SEM-EDX analysis of the sample processed for 3 min in hydrogen plasma reveals a microstructure where Ni, Co, and Fe are dissolved within the Mn-oxide phase, which subsequently solidifies within a dark Mn-silicate matrix.

We ball milled the as-received nodule samples to a fine powder (≤100 μm) to ensure homogeneous chemical composition across all experiments. We then compacted the powder into green pellets, smelted them in an electric-arc furnace under an inert argon atmosphere, and exposed them to a lean hydrogen plasma (Ar–2.5% H_2_) at temperatures exceeding 1700°C, as detailed in Materials and Methods. The smelting process under an inert Ar atmosphere resulted in a mass loss of ~39 wt %, attributed to splashing during arc ignition and thermal decomposition reactions at high temperatures and low oxygen partial pressures. Characterization of the solidified samples using SEM-EDX and XRD revealed that they predominantly consisted of two Mn oxide phases: (Mn, Cr, Fe, Ni, Co)_3_O_4_ (42 wt %) and (Mn, Fe, Ni, Co)O (37 wt %), as shown in Fig. 3A. In addition, metallic Cu in the face-centered cubic (FCC) form is detected, although no macroscopic Cu particles could be physically separated upon breaking or powdering of the solidified sample. SEM-EDX analysis (Fig. 2, D to F) confirmed the presence of metallic Cu in the form of dendritic structures distributed within the oxide matrix, with elemental mapping (Fig. 2E) showing the uniform distribution of Cu dendrites throughout the solidified sample. These observations suggest that during solidification, Cu precipitates as a metallic phase, whereas Ni, Co, and Fe remain partitioned within Mn-oxide phases.

To ensure that the precipitation of pure metallic Cu from this complex multioxide material is a reproducible phenomenon and not an experimental artifact, we repeated the experiments for five times, consistently yielding similar results. We conducted a thermodynamic assessment of the Mn-nodule system using the Thermo-Calc software, with equilibrium calculations performed at 1600°C, corresponding to the temperature used in our furnace experiments (Materials and Methods for details). At this temperature, the system partitions into two phases: an ionic melt and a gas phase (fig. S1B). The ionic melt, which is composed of all the valuable targeted metal oxides (Ni, Co, Cu, and Mn), becomes the primary phase of interest. Figure 2B illustrates the partitioning behavior of Cu from this ionic melt across different phases at various temperatures. At temperatures above 1200°C, Cu remains dissolved in the oxidic liquid phase, and upon cooling to the temperature range of 1100° to 1200°C, it precipitates into a metallic liquid state. Further cooling below ~1100°C results in the precipitation of Cu as a solid metallic phase (FCC), marking a transition from the liquid to the solid state during solidification. These results align well with experimental SEM-EDX observations, where Cu dendritic structures were found throughout the solidified matrix (Fig. 2D), indicating phase transformations during cooling.

The observed anomalous behavior of Cu, precipitating as a metallic phase upon cooling but not forming a separate metallic liquid at higher temperatures, is further examined using Gibbs free energy calculations for the Cu-Mn-O systems (Fig. 2C). The as-received polymetallic nodules contain Mn in a highly oxidized state, specifically Mn^+4^ (as MnO_2_), primarily because the nodules form under highly oxidizing conditions, such as those present at the seafloor (*33*). When the MnO_2_ phase is exposed to the high temperatures present in the furnace (above 1600°C), it undergoes thermal decomposition reactions. The thermal decomposition reactions occurring as MnO_2_ is heated are documented in fig. S1. At temperatures above 1700°C, Mn exists predominantly in the +2-oxidation state as MnO. This reduction of oxidation state from Mn^+4^ (MnO_2_) to Mn^+2^ (MnO) occurs purely through thermal decomposition reactions without the use of any reducing agents (e.g., H). A similar behavior is observed for Cu, which in the original feed material is dissolved in MnO_2_ as CuO with a +2-oxidation state. According to the equilibrium phase diagram of the Cu-O system, CuO also undergoes thermal decomposition above ~1150°C, releasing oxygen to the gas phase and transforming into Cu_2_O (*34*). Upon solidification of this system (molten deep-sea nodules), oxygen redistribution takes place below 1200°C, and MnO is reoxidized to Mn_3_O_4_ through oxygen uptake from Cu_2_O, which results in the precipitation of metallic Cu. This redox reaction can be expressed as MnO + Cu_2_O → 2Cu + Mn_3_O_4_, with the associated Gibbs free energy change (Δ*G*) becoming negative below 1200°C (Fig. 2c), indicating a thermodynamically favorable pathway that aligns with the experimental data. From these observations, it can be inferred that, at temperatures below 1200°C, MnO acts as a reducing agent and reduces Cu_2_O to Cu according to the net chemical reaction (MnO + Cu_2_O → 2Cu + Mn_3_O_4_). This qualifies this reduction process as a displacement reaction in which the more reactive metal oxide MnO reduces the less stable metal oxide Cu_2_O, leading to the oxidation of Mn to a mixed-valence oxide spinel, referred to as hausmannite, Mn_3_O_4_. Conversely, the reaction is reversed at temperatures above 1200°C. These insights explain the chemical driving forces behind the oxygen redistribution and the Mn-rich oxide conditions in these nodules required for Cu precipitation during solidification under inert atmospheres. Understanding this behavior offers a promising approach for the sustainable and efficient extraction of valuable Cu directly from such complex Mn-rich oxide mixtures.

### Phase transformation and reduction mechanisms

**Fig. 4.**
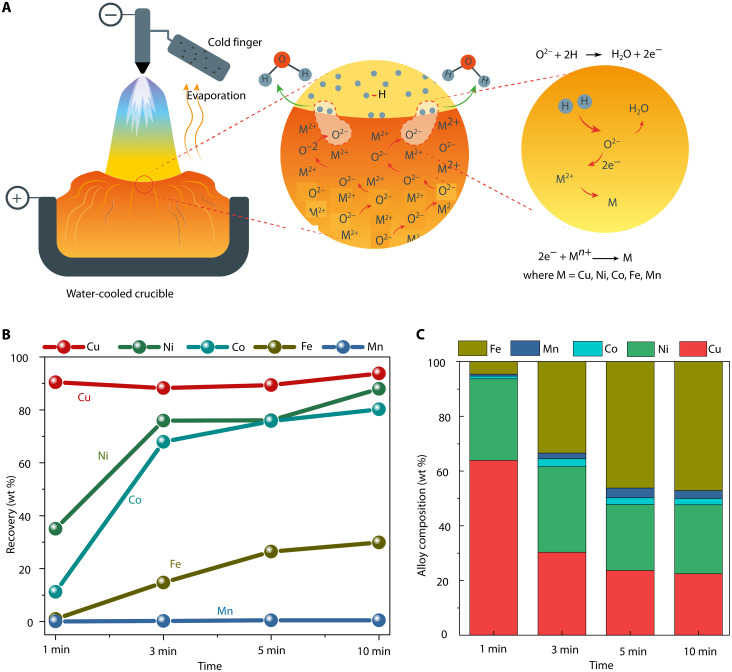
Reduction mechanism and evolution of elemental recovery and alloy composition during hydrogen plasma processing. (**A**) Schematic hypothesis of reduction mechanism: Precipitation of metals is initiated by the removal of free oxygen from the melt at the arc-melt interface. Hydrogen species extract free oxygen, leaving behind two electrons (2e^−^). These electrons react with metal cation (M^2+^) having lowest oxygen affinity within the system, inducing the precipitation of metallic elements as a separate phase. The corresponding ionic reactions are also illustrated. (**B**) Variation of elemental “recovery” (viz., ratio between the weight of metallic element in the alloy obtained and the total amount of element present in the original nodule) with exposure time to hydrogen plasmas (Ar–2.5% H_2_) and (**C**) variation of alloy composition [viz., amount of element (in weight %) present in the alloy] with processing time.

The reduction behavior of the as-received Mn nodules is evaluated for samples exposed to a hydrogen plasma arc for durations of 1, 3, 5, and 10 min. The exposure of polymetallic nodules to hydrogen plasmas resulted in the formation of metallic nuggets, which solidified at the bottom of the sample, mainly driven by the differences in viscosity and density. After solidification, the metallic nuggets could be easily separated from the unreduced oxides/silicate components by mechanical crushing. The unreduced silicate/oxide portion is separated and powdered for XRD analysis to trace the phase transformation and chemical partitioning during the reduction progression, as documented in Fig. 3B. XRD analysis revealed that the metals Ni, Co, and Fe do not precipitate as discrete oxide phases in the solidified samples but instead were dissolved within the Mn-O binary matrix. This behavior aligns with findings reported in the prior literature, indicating that the Mn-O structure can accommodate chemically similar metals such as Fe, Ni, and Co by substitution, enabled by their shared +2-oxidation state and comparable ionic radii with Mn (*28*, *35*). Upon analysis of the Ar-melted sample, the primary Mn-oxide phases identified were FeMn_2_O_4_ (42 wt %) and FeMnO (37 wt %), as illustrated in Fig. 3A. Exposure of this sample to hydrogen plasma for 1 min resulted in the reduction of Mn_3_O_4_ to Mn-containing monoxide (MnO), accompanied by the precipitation of metallic elements, specifically a rich alloy containing as much as 64 wt % Cu and 30 wt % Ni (Fig. 4C).

At elevated temperatures (>1700°C), such as those present inside our furnace, the metals (Cu, Ni, Co, and Fe) precipitate directly in liquid form from the remaining oxidized ionic liquid, subsequently coalescing into a distinct liquid alloy phase. The immiscibility between the metallic liquid and the surrounding oxidic/silicate liquid is thermodynamically driven, primarily due to the large differences in their bonding characteristics. Metallic liquids, dominated by metallic bonds, and oxidic/silicate liquids, governed by ionic and covalent interactions, exhibit high interfacial tension, minimizing the adhesive interactions between them. This results in phase separation to reduce the Gibbs free energy of the system, as mixing these phases would yield a positive enthalpy of mixing (Δ*H*_mix_) with insufficient compensating entropy of mixing (Δ*S*_mix_). Once the metallic liquid droplets nucleate within the oxidic liquid, turbulence induced by the plasma arc further facilitates their collision and coalescence into larger metallic droplets (*31*, *36*–*38*). The high surface tension of the metallic phase further drives coalescence, as minimizing interfacial area between the two immiscible liquids is energetically favorable. This phase separation and coalescence process were directly observed through the viewing port of the furnace. Upon cooling, this metallic phase solidified into an alloy, whose composition is found to be depended on the oxygen removal degree (i.e., extent of reduction) at any particular instance (more details in the next section). The unreduced silicate/oxide portion, following 1 min of plasma exposure, solidified with a phase composition primarily consisting of 36 wt % FeMnSiO_4_ and 62 wt % FeMn-O (Fig. 3A). In this solidified structure, Fe, Ni, and Co were incorporated into the Mn-O crystal network, substituting Mn sites to form solid solution phases within the Mn-O matrix, as reported in the literature (*13*, *26*). This FeMn-O phase is a key metal-dissolution phase, which acts as a source for further metal precipitation as reduction proceeds (Fig. 3A). This is further corroborated by SEM-EDX elemental mapping analysis of the oxide/silicate portions, which confirmed that Ni and Co consistently remain dissolved within the Mn-O phase throughout the entire reduction process, without partitioning into discrete oxide phases (Fig. 3C), which shows elemental mapping of sample reduced for 3 min. Prolonged exposure to hydrogen plasma results in the depletion of the FeMn-O phase, accompanied by the precipitation of metallic alloys. Concurrently, the slag (unreduced silicate/oxide part) composition becomes progressively enriched with the FeMnSiO_4_ phase. This enrichment occurs because of the consumption of the FeMn-O phase for metal extraction (Fig 3A).

XRD analysis provides valuable insights into phase evolution within solidified samples during the reduction sequence; however, the primary reduction reactions occur in the molten state. Therefore, understanding the compositional evolution of molten species under reducing conditions is essential to delineate the reduction mechanisms that control metal precipitation. For this purpose, the ionic liquid behavior of this system is modeled using Thermo-Calc software package 2024A, coupled with the TCOX10 and SSUB5 database. Upon melting, the complex crystal structure exhibited by polymetallic nodules is broken down and is transformed into an ionic liquid (Fig. 3B), which shows the compositional evolution of this ionic melt upon oxygen removal. The main constituents of this ionic liquid are Cu^1+^, Ni^2+^, Fe^2+^, Co^2+^, Mn^2+^ (at sublattice 1), MnO_1.5_, CuO, FeO_1.5_, O^2−^, and SiO_4_^4−^ (at sublattice 2). The free oxygen (O^2−^) contribution arises mainly from the breakdown of the Fe-Mn-O halite phase, which hosts the critical metals dissolved within it. The detailed explanation about the sublattice model can be found in (*39*). Within the ionic melt, oxygen partitions into various species, among which free oxygen species (O^2−^), are the most reactive with hydrogen plasma species, forming water (H_2_O) and leaving the melt as vapor. The continuous removal of O^2−^ from the melt drives the precipitation of metals within the system. Oxygen removal primarily occurs at the arc-melt interface, where temperatures are estimated to reach ~2000°C (*9*, *40*, *41*) higher than the bulk melt temperature (above 1600°C). These elevated temperatures at the interface contribute to the rapid kinetics of the hydrogen plasma smelting reactions. At the interface, hydrogen plasma species (H, H^+^, and H_2_) interact with O^2−^ through the following reaction: (O^2−^ + 2H/2H^+^/H_2_ → H_2_O + 2e^−^). The water vapor formed exits the melt, while the two electrons released during this reaction reduce nearby metal ions (Cu^2+^, Cu^1+^, Ni^2+^, Fe^2+^, and Mn^2+^) to their metallic states via the following reaction: (2e^−^ + M*^n+^ →* M; where M = Cu, Ni, Co, Fe, and Mn). Metal precipitation is thermodynamically favored for ions with lower oxygen affinity, with Cu precipitating first, followed sequentially by Ni, Co, Fe, and Mn. A simplified schematic of this process is depicted in Fig. 4A, illustrating the oxygen removal and subsequent sequence of metal precipitation within the system.

The metallic alloys obtained at various stages of reduction (1, 3, 5, and 10 min) assumed a spherical geometry, characteristic of molten-state processing, as shown in Fig. 1C and fig. S2. After 1 min of reduction, some metallic spheres appeared Cu colored, indicating compositional nonuniformity among the metallic alloys obtained after 1 min of process. This nonuniformity is further confirmed by SEM-EDX analysis, and to homogenize the composition, the metallic nuggets were remelted under inert atmosphere (fig. S2). The composition of metallic alloys after remelting is therefore analyzed by probing their cross sections using SEM-EDX. Their compositional evolution is documented for different hydrogen plasma exposure times as shown in Fig. 4D. During the initial stages of reduction, the alloy is predominantly enriched in Cu (64 wt %) and Ni (30 wt %), attributed to the lower oxygen affinity of these elements, which facilitates their preferential reduction and coprecipitation as a metallic phase. As the exposure time increases, the alloy is progressively enriched in Fe. After 10 min of exposure, the alloy composition evolves to 47 wt % Fe, 22 wt % Cu, 25.1 wt % Ni, 2.3 wt % Co, and 3 wt % Mn. Notably, within just 1 min of exposure, ~93 wt % of the total Cu present in the initial Mn nodules is reduced to the metallic form, forming a Cu-rich alloy. The recovery of Cu remains consistently high (~93 wt %) with extended exposure, as shown in Fig. 4B, which tracks the elemental recovery as a function of exposure time.

## DISCUSSION

In this study, we provide evidence of the direct one-step sustainable alloy formation from polymetallic nodules via rapid reduction using high-energy hydrogen plasma species. The electrified reduction process is characterized by fast kinetics (within minutes), the production of water as a by-product, and the absence of any direct CO_2_ emissions (as no fossil reactants are used), distinguishing it from conventional processing routes. It is also observed that pure Cu precipitates spontaneously from the system during solidification when melted in an inert atmosphere for 2 min, without the need for the use of an added reducing agent. This finding is particularly important for the processing of the Mn-rich polymetallic nodules, as it enables the selective recovery of pure Cu from nodule samples, which have the reductant already “onboard” (MnO). Given the rising demand for Cu required for coming global electrification, this method represents a critical additional advantage, offering a simplified and sustainable approach to Cu extraction. Although the current study focuses on a single-step HPSR of the entire feed material, this observation of pure Cu precipitation suggests an additional potential process strategy. In this approach, Cu may be selectively removed by initially melting the feed under inert conditions, enabling the precipitation of metallic Cu. This can be physically separated from the oxide residue, yielding a Cu-depleted residue. The remaining material, enriched in Ni, Co, and Fe, can then undergo HPSR to form a Cu-free alloy. Considering the declining grades of Cu ores and increasing energy demands of multistep carbothermal processing for Cu extraction from terrestrial deposits, this method presents a sustainable alternative. Using polymetallic Mn-rich nodules as a source of Cu through this process, without using reducing agents (C or H_2_), could affect the global Cu supply chain in the future.

During the reduction process, the product comprises an alloy and an unreduced silicate/oxide residue within the crucible. In the later stages of reduction, evaporation from the molten sample is observed through the viewing window. Analysis using a cold finger (electrostatic filter) (details provided in the Supplementary Materials section “Mechanisms of Mn evaporation”) identified the evaporated species as Mn rich, which condenses as dust. XRD and SEM-EDX analyses confirmed that this dust predominantly consists of MnO (76 wt %). The mechanisms underlying Mn evaporation are detailed in the Supplementary Materials section “Mechanisms of Mn evaporation.” The mechanism of Mn removal observed in this study is consistent with previous literature findings (*42*) on Mn removal from Ni melts using hydrogen plasma treatments. In this study, we reveal the formation of three distinct products: MnO-rich dust, a Fe-Mn-Si-O–dominated slag, and an alloy enriched in critical metals such as Cu, Ni, and Co. Quantitative mass-balance analysis shows that FeMnSiO_4_ hosts 22.8 wt % of the initial Mn, FeMnO 9.0 wt %, and the metallic alloy 0.4 wt %. The remaining 67.8 wt % of Mn resides in the volatilized fraction, predominantly as MnO in the gas/dust phase. MnO is particularly valuable as a precursor for battery materials, as it can be directly refined with minimal processing. In contrast, the FeMnSiO_4_ slag is less suitable for battery applications due to the chemical stability of the silicate structure, which makes Mn liberation challenging. However, this slag can serve as a precursor for the production of FeMnSi alloys, as demonstrated by Sommerfeld *et al.* (*28*). By recovering MnO from the dust for battery applications and using the silicate slag for alloy production, the process maximizes resource efficiency while minimizing waste and energy consumption.

**Fig. 5.**
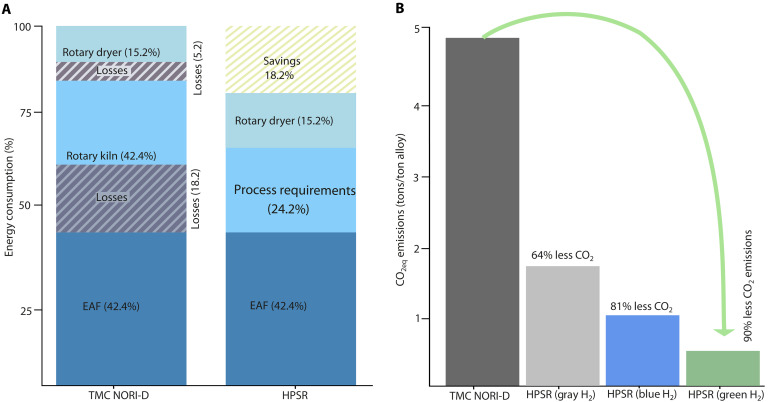
Energy consumption and CO_2_ emission comparison between RK-EF–based TMC NORI-D and HPSR processing routes. (**A**) Comparison of input energy consumption across different processing steps in the RK-EF–based TMC NORI-D process and HPSR. The illustration highlights that energy used in rotary dryers and rotary kilns in the RK-EF process is not fully used for process requirements, leading to losses of ~15.2 and 18.2% to the environment, respectively (represented by maroon strips). In contrast, the direct processing of dried ore in the HPSR route mitigates these losses, enabling energy savings of up to 18% (depicted by light green strips in the HPSR column). (**B**) Comparison of CO_2_ emissions between the TMC NORI-D route and HPSR. HPSR has the potential to reduce emissions by up to 90% if the hydrogen used is produced as green hydrogen and by up to 81 or 64% if the hydrogen is produced via steam methane reforming with carbon capture (blue hydrogen) or without carbon capture (gray hydrogen), respectively. In both the cases (TMC NORI-D and HPSR), the electricity is assumed to be derived from renewable sources.

The current commercial technologies suggested and developed for processing such polymetallic nodules, such as, for example, implemented by TMC in the NORI-D project, rely on the RK-EF route, a multistep pyrometallurgical process that contributes to CO_2_ emissions. The use of carbon-based compounds as heat sources and reductants produces about 4.84 tons of CO_2eq_ per ton of alloy (*29*). Approximately 90% of these emissions are attributed to the pyrometallurgical stages, particularly to the carbon-based smelting and reduction processes. The electricity used in the process comes from renewable sources and hence does not contribute to overall CO_2_ emissions, and all the emissions reported are stemming solely from the use of carbon-based compounds. In contrast, HPSR offers a completely carbon-free alternative by leveraging hydrogen plasma species (H, H^+^, and H_2_) as reductants to produce metallic alloys (containing Ni, Cu, and Co), MnSiO4 slag, and MnO, with water as the only by-product. A fundamental feature of the HPSR method is that it allows combining and balancing the reducing effects of electrical and chemical energy using sustainably generated electrons to excite H_2_ molecules into an excited plasma state, thereby replacing costly chemical driving forces with sustainable electronic contributions. This leverages the electrification of metallurgical synthesis processes, a step that otherwise represents a fundamental challenge. Drying of the as-received material in this process can be conducted using the heat from electric-arc furnace off-gas containing unreacted H_2_ and H_2_O, supplemented by partial combustion of H_2_ if required (*43*). Preliminary calculations indicate that the HPSR route could reduce CO_2_ emissions by up to 90% compared to the RK-EF process (Fig. 5B) (provided the energy and hydrogen are procured from renewable sources), eliminating the reliance on carbon-based reductants and associated emissions. The total amount of hydrogen required to produce 1 ton of the alloy through this method is around 128 kg; the detailed calculations are presented in the Supplementary Materials section “Theoretical estimations of hydrogen requirement.” The direct processing of Mn-rich polymetallic nodules without a calcination step through HPSR offers a more energy-efficient alternative. The calcination step involves energy losses, including heat loss to the furnace body, dust emissions, and material transfer losses, amounting to up to 18% of total energy consumption (Fig. 5A). By bypassing calcination and directly processing dried nodules in a single smelting-reduction step in the electric-arc furnace, the HPSR method holds potential to eliminate such losses, enhancing energy efficiency (Fig. 5A) and simplifying the process for greater sustainability and cost-effectiveness.

All in all, the HPSR process offers a sustainable and energy-efficient alternative for processing Mn nodules, provided they are mined using environmentally responsible methods. This study highlights the environmental impacts of metal production, specifically CO_2_ emissions, associated with processing these nodules sustainably. Evaluating the potential impacts of producing metals from Mn nodules is crucial to inform decisions regarding their approval for mining and to compare the overall environmental effects of this approach with those of terrestrial resource extraction, which often entails deforestation and biodiversity loss. While HPSR offers substantial potential to decarbonize metal production, its large-scale deployment will hinge on the cost and availability of green hydrogen (*44*, *45*). Current global output produced mainly by water electrolysis powered by renewable electricity remains limited and costs about US$4 to US$12 kg^−1^, depending on location, electrolyzer technology, and electricity prices (*46*–*50*). Even under nonideal supply conditions, our analysis shows that HPSR can reduce CO_2_ emissions by ~64% with gray hydrogen, ~81% with blue hydrogen, and ~90% with green hydrogen (Fig. 5). Projections suggest that continued deployment, falling renewable-energy costs, and improved electrolysis technology could drive green hydrogen prices below US$2 to US$3 kg^−1^ by 2030 (*45*, *51*), alongside expanded infrastructure for compression, storage, and transport. Parallel efforts to develop natural hydrogen and other emerging low-carbon sources may further strengthen supply (*52*). Early industrial adoption including processes such as HPSR can create the demand pull needed to catalyze investment and accelerate the build out of the green-hydrogen supply chain, thereby reinforcing the pathway to large-scale, low-carbon metal extraction.

## MATERIALS AND METHODS

### Material preparation

The material used in this study consists of deep-sea polymetallic nodules, sourced specifically from the CCZ. These nodules, appearing in irregular, potato-like shapes and sizes, were ball milled to a uniform particle size of ~100 μm to ensure consistency. The elemental and mineralogical compositions were analyzed using inductively coupled plasma optical emission spectrometry and XRD, with detailed results presented in extended tables S1 and S2, respectively.

### Reduction experiments

The processed ore powders were compacted into green pellets under a uniaxial load of 20 × 10^6^ Pa. All pellet batches used for reduction experiments were weighed with an accuracy of ±0.1 g, and recovery calculations for each run were based on the precise initial mass to ensure consistency and reproducibility of the data. The pellets were placed on a water-cooled Cu hearth inside a conventional arc melting furnace, equipped with a 6-mm-diameter tungsten electrode. The 18-liter furnace chamber is purged with an Ar–2.5% H_2_ gas mixture at a pressure of 900 mbar. Reduction is initiated via a 200-A arc plasma between the electrode and the pellets, enabling simultaneous melting and reduction over a 1-min period. During the process, the voltage was recorded to remain within the range of 20 to 24 V. After each cycle, the arc is shut off, the sample solidified, and the atmosphere is refreshed with new gas to continue reduction in successive stages. The process is carried out in batches and does not involve a continuous flow of gasses.

### Phase analysis

Following solidification, small cross sections are extracted for microstructural analysis. The remaining material is manually fractured or broken to recover millimeter-scale metallic nodules from the surrounding silicate matrix. Powdered samples are prepared by grinding to <75 μm and homogenizing to ensure representative sampling. XRD patterns are collected on a Bruker D8 Advance diffractometer using Cu-Kα radiation (λ = 1.5406 Å) operated at 40 kV and 40 mA. Data were acquired over a 2θ range of 20° to 120° with a step size of 0.02° and a counting time of 1 s per step. Instrumental broadening was determined using a National Institute of Standards and Technology Al_2_O_3_ + Si standard measured under the same conditions. Quantitative phase analysis is conducted using Rietveld refinement implemented in Bruker TOPAS Version 5.0. The refinement protocol comprised the following:

1) Background fitting with a third-order Chebyshev polynomial.

2) Structural parameters including lattice constants, zero shift, and peak asymmetry corrections.

3) Microstructural broadening modeled via crystallite size (*L*).

4) Preferred orientation corrections using sixth-order spherical harmonics combined with the March-Dollase function.

These parameters are chosen to account for instrumental and sample-related effects, providing a robust fit to the measured diffraction patterns. The weighted profile *R* factor (Rwp ≈ 10) indicated good agreement between observed and calculated patterns.

### Microstructural characterization

Selected samples are prepared through standard metallographic techniques, including grinding, polishing, and final polishing with colloidal silica, oxide polishing suspension (OPS). Imaging and elemental analysis were performed using a Zeiss Merlin high-resolution scanning electron microscope equipped with EDX.

### Thermodynamic simulations

Equilibrium simulations are carried out using Thermo-Calc, applying the TCOX10 Metal Oxide Solutions Database for the liquid phase and the SSUB5 SGTE Substances Database for the gas phase, which accounts for both metallic and oxide vapors. The liquid phase is modeled via a two-sublattice approach involving species such as (Fe^2+^, Mg^2+^, Ni^2+^, Cu^+1^, Mn^2+^, Co^2+^, Si^4+^) and (O^2−^, SiO_4_^4−^, VA^−^, FeO_1.5_, MnO_1.5_, CoO_1.5_, CuO, SiO_2_), maintaining charge neutrality through variable site occupancy. Simulations assumed a 10-g ore input with the composition provided in table S1, held at 1600°C, and exposed to varying flow rates of Ar–2.5% H_2_ under constant pressure (0.9 × 10^5^ Pa). Element partitioning is allowed across all phases.

### Energy and CO_2_ emission assessment

The energy analysis compares the HPSR process with the conventional RK-EF route used in the TMC NORI-D process. Because of scale-related inefficiencies in the laboratory-scale HPSR system (e.g., water-cooled hearth), direct energy extrapolation was avoided. Instead, both routes were compared on the basis of their theoretical thermodynamic energy requirements, which remain similar. Energy distribution data for RK-EF were taken from (*53*), and process integration benefits of HPSR (e.g., elimination of calcination and transfer losses) were included to estimate potential energy savings. Full methodological details are available in the Supplementary Materials section titled “Energy and CO_2_ emission comparisons.” CO_2_ emissions for the RK-EF route are based on published life cycle assessment data (*29*), accounting for mining and primary extraction stages (drying, calcining, melting, and reduction). For HPSR, emissions from mining are calculated using nodule grades and recovery rates, while emissions from primary extraction are assumed to be zero due to the use of green hydrogen and renewable electricity. In this study, we classify hydrogen according to its production pathway and carbon footprint: “Green hydrogen” refers to hydrogen produced by electrolysis using renewable electricity; “blue hydrogen” refers to hydrogen produced primarily by steam methane reforming with carbon capture and storage (CCS); and “gray hydrogen” refers to hydrogen produced by the same reforming process without CCS. The emissions from the process are estimated using average CO_2_ intensities of 2.3 kg CO_2_/kg H_2_ for blue hydrogen and 10 kg CO_2_/kg H_2_ for gray hydrogen, as reported in (*54*, *55*). Total CO_2_ emissions are then calculated by multiplying the total hydrogen consumed in the process by the respective CO_2_ footprint of each hydrogen source. Emissions per kilogram of alloy are computed for both processes, and the reduction potential of HPSR was quantified accordingly. Full methodological details are available in the Supplementary Materials section titled “Energy and CO_2_ emission comparisons.”
