# Supplementary material for: Low-waste, single-step, sustainable extraction of critical metals from deep-sea polymetallic nodules
Source: Sci Adv. 2025 Nov 21;11(47):eaea1223. doi: 10.1126/sciadv.aea1223 (PMC12637279; doi:10.1126/sciadv.aea1223)
Supplement: Supplementary file 1 — Supplementary Text Figs. S1 to S5 Tables S1 to S4 [file sciadv.aea1223_sm.pdf]

Science Advances
AAAS

Supplementary Materials for

Low-waste, single-step, sustainable extraction of critical metals from deep-sea polymetallic nodules

Ubaid Manzoor *et al.*

Corresponding author: Ubaid Manzoor, u.manzoor@mpie.de

Sci. Adv. **11**, eaea1223 (2025)
DOI: 10.1126/sciadv.aea1223

This PDF file includes:

Supplementary Text
Figs. S1 to S5
Tables S1 to S4

Supplementary Text

Mechanisms of Mn evaporation

The reduction of Mn-nodules using hydrogen plasmas at high temperatures results in dust formation and evaporation phenomena, particularly during the ignition and later stages of the process. To study the chemical and phase composition of the resulting dust, a cold finger or electrostatic filter setup was employed. This setup consists of a copper sheet connected to an electrode, positioned either directly above the sample (Fig. 4a). The cold finger acts as a condensation surface for vaporized species due to its low temperature, facilitating the collection of condensed material during the reduction process. The more details about the setup can be found in the previous study by Büyükuslu et.al (*41*).

Experiments conducted under a hydrogen-containing plasma atmosphere (Ar-2.5% H_2) with a 12-minute exposure time produced a visible dust layer on the cold finger, indicating condensation events. Compositional analysis of this layer was carried out using scanning electron microscopy with energy-dispersive X-ray spectroscopy (SEM-EDX) and X-ray diffraction (XRD). SEM-EDX results (Fig. S3 (B-C)) revealed a composition predominantly of Mn and O, with stoichiometry corresponding closely to the MnO phase (Fig. S3D). This observation was further corroborated by XRD analysis (Fig. S3B), which showed the condensed dust is enriched with MnO (76 wt.%).

Comparative experiments conducted in an inert plasma atmosphere (100% Ar) revealed distinct differences in evaporation behavior. Evaporation was primarily observed during the initial ignition stage only, after which it diminished, resulting in a relatively thin layer of deposits on the cold finger. Compositional analysis of these deposits, performed via XRD, confirmed the absence of MnO, with the condensed material being enriched with CuO and ZnO phases. These findings highlight the critical role of the furnace atmosphere in influencing Mn evaporation during the reduction process. Specifically, the reducing hydrogen-containing atmosphere (Ar-2.5% H_2) facilitates the removal of Mn from the melt, as evidenced by dominant presence of Mn-O in the cold finger deposits, contrasting sharply with the behavior observed under an inert atmosphere.

To explore the mechanisms of Mn evaporation, thermodynamic simulations were performed using the Thermo-Calc software package (version 4.0) coupled with the TCOX10 database for ionic melts and the SSUB5 database for gaseous species. These simulations were conducted for equilibrium conditions at temperature of 1600°C, corresponding to bulk melt temperatures. The results demonstrated that upon exposure to hydrogen, oxygen removal from the melt occurs as

water vapor, driving the precipitation of metals. Equilibrium partitioning data of Mn (Fig.S4A) reveals that starting from oxidic liquid, Mn transitions into the metallic liquid phase, saturating at approximately 2 wt.% Mn, while progressively partitioning into the gaseous phase with continued hydrogen exposure. It was further observed that in the gaseous phase (simulated with SSUB5), Mn exists as both elemental Mn vapor and manganese hydride (MnH) vapor.

Based on these observations, we hypothesize that Mn evaporation occurs through the following steps:

1. Mn-O is reduced to metallic Mn, which subsequently alloys with Cu, Ni, Co, and Fe in liquid state.
2. At high temperatures at the arc-melt interface (>2000°C, near Mn's boiling point of 2061°C), metallic Mn begins to partition into gaseous phase (Mn (l) → Mn (g)), owing to its high partial pressure at such temperatures.
3. In the arc region, Mn vapor undergoes a reaction with hydrogen plasma species to form manganese hydride (MnH). This process reduces the partial pressure of Mn vapor above the melt, creating a thermodynamic driving force that accelerates the partitioning of Mn from the melt to the vapor phase. As a result, Mn continuously transitions into the vapor phase during the exposure, maintaining the dynamic equilibrium dictated by hydride formation.
4. Upon exiting the arc region, MnH dissociates into Mn and H. The released Mn subsequently reacts with water vapor, which is generated during the reduction process, leading to its oxidation into MnO. This MnO was observed as condensate dust deposited on the cold finger.

Similar findings have been reported in the literature (*42*), highlighting the use of hydrogen plasma to facilitate the removal of Mn dissolved in Ni and Fe melts. These studies demonstrated that Mn removal occurred exclusively in a hydrogen plasma environment and was absent under inert atmospheric conditions. The results further concluded that the formation of manganese hydride (MnH) in the gas phase is the primary mechanism driving the continuous transfer of Mn from the melt to the gaseous phase. However, it should be noted that the proposed existence of manganese hydride is inferred solely from thermodynamic calculations and the observed behavior of the system; no direct experimental evidence for MnH formation was obtained in this study.

These observations, summarized schematically in Fig. S5, it provides a comprehensive understanding of the complex interplay between reduction reactions, high-temperature evaporation, hydride formation, and subsequent oxidation that governs Mn evolution during the reduction of Mn-nodules. For simplicity, only Mn species are shown in the schematic, with the different steps highlighted by arrows indicating the corresponding transitions. Step 1: Reduction of Mn^{2+} to Mn by hydrogen plasma species. Step 2: Evaporation of Mn from the melt into the vapor phase due to its high partial pressure. Step 3: Formation of MnH_x in the vapor phase, which reduces the vapor pressure of Mn. Step 4: Exit of MnH from the arc, followed by its dissociation into Mn and H_x.

Theoretical estimations of hydrogen requirement:

Nodules Basis: 1 ton of nodules, metal masses based on ICP-OES results presented in Table S1.

Recovery factors: Ni (88%), Cu (94%), Co (80%), Fe (67%) (experimental values)

Reducing agent: hydrogen (H)

Atomic weights: O = 16 g/mol, H = 1 g/mol.

The amount of hydrogen required is calculated based on the quantity of metal extracted and the corresponding amount of oxygen that must be removed from the metal oxides to form the metallic phase. This is determined using stoichiometric relationships. Oxygen released through thermal decomposition is assumed not to consume hydrogen. Based on both theoretical and experimental observations, MnO_2 is assumed to decompose thermally to MnO, and Fe_2O_3 is assumed to decompose to Fe_3O_4. The oxidation states used in the calculations are derived from Thermo-Calc simulations, which reflect the predominant oxide phases at high temperatures. Equivalent oxygen quantities are assigned to each metal cation according to these oxidation states to estimate the hydrogen demand. Based on the Mn mass balance, Mn partitioning is as follows: $FeMnSiO_4$ contains 22.8 wt.% of the initial Mn, FeMnO 9.0 wt.%, the alloy 0.4 wt.%, and the evaporated/dust fraction accounts for the remaining 67.8 wt.%. This means only 0.4 wt.% of Mn partitions into metallic phase

Thermodynamic calculations suggest that in the molten state at the processing temperature (~1700 °C), metals exist in the following oxidation states, forming their respective oxides:

- Ni: Ni^{2+} → equivalent to NiO
- Cu: Cu^{+} → equivalent to Cu_2O

- Co: Co^{2+} → equivalent to CoO
- Fe: Fe^{2+} and Fe^{3+} → forms mixed oxide Fe_3O_4 (magnetite: $FeO·Fe_2O_3$)

The general hydrogen reduction reaction in the molten state is:

$$M^{2+} + O^{2-} + 2H \rightarrow M + H_2O$$

Specific reactions are:

- $Ni^{2+} + O^{2-} + 2H \rightarrow Ni + H_2O$
- $Cu_2O + 2H \rightarrow 2Cu + H_2O$ (Cu^+ oxide)
- $Co^{2+} + O^{2-} + 2H \rightarrow Co + H_2O$
- $Fe_3O_4 + 8H \rightarrow 3Fe + 4H_2O$ (mixed Fe^{2+} and Fe^{3+})

For each metal oxide, the reduction reactions, molar calculations, and mass of hydrogen required are given below.

Nickel (Ni) – as NiO

- Reaction: $NiO + 2H \rightarrow Ni + H_2O$
- Mass Ni: 12,000 g
- Moles Ni: 204.4 mol, Moles O: 204.4 mol
- Moles H needed: 408.8 mol
- Mass H: 0.4088 kg
- Adjusted for recovery (88%): 0.3597 kg H

Copper (Cu) – as Cu_2O

- Reaction: $Cu_2O + 2H \rightarrow 2Cu + H_2O$
- Mass Cu: 10,000 g
- Moles Cu: 157.4 mol, Moles Cu_2O: 78.7 mol, Moles O: 78.7 mol
- Moles H needed: 157.4 mol
- Mass H: 0.1574 kg
- Adjusted for recovery (94%): 0.148 kg H

Cobalt (Co) – as CoO

- Reaction: $CoO + 2H \rightarrow Co + H_2O$
- Mass Co: 1,200 g
- Moles Co: 20.37 mol, Moles O: 20.37 mol

- Moles H needed: 40.74 mol
- Mass H: 0.04074 kg
- Adjusted for recovery (80%): 0.0326 kg H

Iron (Fe) – as Fe_3O_4

- Reaction: $Fe_3O_4 + 8H \rightarrow 3Fe + 4H_2O$
- Mass Fe: 66,000 g
- Moles Fe: 1181.2 mol, Moles Fe_3O_4: 393.73 mol, Moles O: 1574.9 mol
- Moles H needed: 3149.8 mol
- Mass H: 3.15 kg
- Adjusted for recovery (67%): 2.11 kg H

Manganese (Mn) – as MnO (based on findings from section "mechanisms of Mn evaporation")

- MnO_2 is thermally decomposed to MnO.
- Subsequent reaction: $MnO + 2H \rightarrow Mn + H_2O$
- At high temperatures, Mn in liquid form transitions to vapor and reacts with hydrogen forming manganese hydride (MnH), which decomposes outside the arc as:

  $MnH \rightarrow Mn + H$
- This Mn, upon encountering water vapor, reforms MnO and releases hydrogen:

  $Mn + H_2O \rightarrow MnO + 2H$
- Due to this cyclic behavior and primary thermal decomposition to MnO, no net hydrogen consumption is attributed to Mn.
- In the alloy there is 2.9 wt.% Mn, which means in 1 ton of alloy contains 29 kg of Mn. The theoretical amounts of hydrogen required are 1.06 kg.

Total Hydrogen Required:

Hydrogen required: 0.360(Ni) + 0.148(Cu)+ 0.033(Co)+ 2.110(Fe) + 0.022 (Mn) = **2.67 kg/ton nodule**

This amount is equivalent to **128.16 kg H_2/ton alloy**

Considering a large-scale setup similar to a typical electric arc furnace with a feed capacity of 250–350 tons, and given TMC NORI-D's estimated batch capacity of approximately 225 tons, the

projected hydrogen requirement would be approximately 667–934 kg per batch. The hydrogen requirement reported here corresponds solely to the stoichiometric quantity necessary for the chemical reactions. In practice, the total hydrogen feed will exceed this amount and will depend on reactor efficiency and operating conditions. Nevertheless, unreacted hydrogen can be recovered from the off-gas using well-established technologies, such as those already implemented in direct reduction iron (DRI) reactors (*43*). Consequently, only the hydrogen converted to water should be considered as irreversibly consumed, while the unreacted fraction is not regarded as a loss because it can be efficiently recovered and recycled to the reactor.

Energy and CO_2 emission comparisons

Energy comparisons

The energy comparison presented here is intended to provide a realistic perspective on the potential large-scale implementation of HPSR technology and the energy savings it could offer relative to the industrial RK–EF route used in the TMC-NORI-D process. In our laboratory-scale HPSR reactor, the use of a water-cooled copper hearth leads to heat extraction from the furnace, which does not reflect the design of large-scale systems where ceramic-based hearths are typically employed to minimize thermal losses. Consequently, the electrical energy input measured in the lab is not representative of actual process energy consumption, as a large portion is diverted to cooling rather than driving the process itself. Moreover, laboratory energy values often do not scale linearly due to differences in heat retention, system geometry, and reaction kinetics at scale. To avoid speculative extrapolations, we instead benchmark the HPSR route directly against the commercial RK–EF process. This comparison is scientifically valid because the theoretical thermodynamic energy requirement i.e., the energy needed to heat, melt, and chemically reduce the ore remains essentially the same regardless of the technology used. The only distinction arises from the heat of reaction, which differs between carbon-based (RK–EF) and hydrogen-based (HPSR) reductants.

According to prior literature (*9*, *30*), The difference in energy consumed for chemical reduction reactions during the processing of low-grade Ni-laterites (∼1.26 wt.% Ni) at 1600 °C via the RK–EF route versus the HPSR route is less than 2% of the total energy input(*9*, *30*). This indicates that, for both RK–EF and HPSR processes applied to low-grade Ni-laterite ores, the overall thermodynamic energy requirement, including heating, melting, phase transformations, and reduction enthalpies is essentially equivalent. Specifically, the contribution from reaction

enthalpy differs by only ~2% of the total energy consumed, affirming that the key thermodynamic demands are comparable across both technologies.

Given that polymetallic nodules have similar Ni content (~1.3 wt.%) to low-grade laterite ores (1.2 wt.%), the partitioning of energy between melting and reduction stages is expected to be comparable. As such, this comparison focuses on process integration as the critical differentiator: while RK–EF based TMC NORI-D relies on multi-stage material transfers and separate reactors for calcination, smelting, reduction and refining, HPSR consolidates all steps into a single furnace. This design inherently avoids heat losses from inter-stage transfers and enables potential energy savings, as detailed in the following paragraph. Additional HPSR benefits, such as faster kinetics, elimination of external refining steps, and impurity removal during smelting are acknowledged but excluded from this energy comparison to maintain a conservative and speculation-free analysis.

According to Liu et al. (*53*), the energy distribution in the RK-EF route is characterized by the following breakdown: rotary dryers account for 15.2% of the total energy consumption, while both rotary kilns and Electric Arc Furnaces (EAF) consume 42.4% each. The primary functions of rotary kilns and dryers involve the removal of moisture and chemically bound water, heating the ore and partially reducing it. This represents the minimum energy required for optimal process functionality. Energy dissipation occurs through various channels, including losses from flue gases, dust dispersion, and furnace body inefficiencies. Notably, 15% loss is incurred during material transfer between rotary kilns and EAF. The total losses occurring during the calcination stage account for approximately 18.2% of the total energy required for the entire process, as detailed in Table S3.

Direct processing of dried nodules via HPSR can theoretically eliminate all calcination-related losses, improving energy efficiency by up to 18.2%. This advancement supports energy conservation and sustainable extraction of critical metals from Mn-nodules. Additionally, hydrogen plasma reduction offers faster kinetics than carbothermic reduction and yields a purer final product, eliminating the need for impurity removal (Si, P, C, S, etc.). This streamlined process shortens processing time compared to the conventional RK-EF based TMC NORI-D route, further enhancing energy savings.

CO_2 emissions

The current commercial processing technology for Mn-nodules, implemented in the TMC NORI-D project, is based on the RK-EF method, an energy-intensive, multi-step pyrometallurgical

process that relies heavily on carbon-based compounds as both heat sources and reductants. As a result, it generates CO_2 emissions. A comprehensive, ISO 14040/14044-compliant life cycle assessment (LCA) has been conducted for the TMC-NORID process(*29*), covering all stages from mining to final product. This large-scale, independently verified study serves as a robust benchmark for environmental impact assessment for deep sea polymetallic nodules processed this method.

In contrast, the HPSR process replaces carbon-based inputs with renewable electricity and green hydrogen, effectively eliminating CO_2 emissions from primary extraction. Given the early stage of HPSR development, this study focuses on the key differentiator: the avoidance of emissions from the primary extraction phase. Using the TMC-NORID LCA as a conservative baseline, the comparison offers credible insight into the emission reduction potential of HPSR if deployed at scale. This targeted approach avoids speculative modeling while clearly illustrating the decarbonization advantages of transitioning to cleaner energy and reductant sources in future metal extraction processes.

For the comparison following data was used from the reported LCA(*29*). In the LCA, the alloy composition is reported only in terms of the equivalent amounts of critical metals (Cu, Ni, Co, and Mn). To maintain consistency and for comparison purposes only, we also show the HPSR alloy in terms of these critical metals, demonstrating that the equivalent alloy compositions are comparable. These equivalent compositions are not used in the CO_2 calculations; for the emission estimates, only the experimental recoveries and the composition of deep-sea nodules are used to determine metal yields.

TMC NORI-D produced alloy

Composition (% by weight): Ni = 54.6%, Cu = 41.3%, Co = 4.1%

Recoveries from nodules: Ni = 95%, Cu = 86%, Co = 77%

Nodules Requirement:

42 kg of nodules required per 1 kg of alloy

Emission Factors:

Mining = 0.00969 kg CO_2 per kg of nodules = 0.406 kg CO_2 per kg alloy

Primary extraction = 4.43 kg CO_2 per kg alloy

Total emissions = 0.406 + 4.43 = 4.84 kg CO_2 per kg alloy

HPSR produced alloy

Equivalent alloy Composition (% by weight): Ni = 50.42%, Cu = 44.8%, Co = 4.6%

Nodules Grades and Recoveries:

Ni: Nodules grade = 1.2% (0.012), Recovery = 88% (0.88)

Cu: Nodules grade = 1.0% (0.01), Recovery = 94% (0.94)

Co: Nodules grade = 0.12% (0.0012), Recovery = 80% (0.80)

Step-by-Step Calculation for CO_2 emissions

General Formula:

$$\text{Nodules required (kg)} = \frac{\text{Metal in alloy (kg)}}{(\text{Ore grade} \times \text{Recovery efficiency})}$$

Nickel (Ni):

Metal in alloy = 0.5042 kg, Nodules grade = 0.012, Recovery = 0.88

$$\text{Nodules required} = \frac{0.5042}{(0.012 \times 0.88)} = 47.75 \text{ kg nodules}$$

Copper (Cu):

Metal in alloy = 0.448 kg, Nodules grade = 0.01, Recovery = 0.94

$$\text{Nodules required} = \frac{0.448}{(0.01 \times 0.94)} = 47.66 \text{ kg nodules}$$

Cobalt (Co):

Metal in alloy = 0.046 kg, Nodules grade = 0.0012, Recovery = 0.80

$$\text{Nodules required} = \frac{0.046}{(0.0012 \times 0.80)} = 47.92 \text{ kg nodules}$$

Governing value:

The maximum value governs total nodules requirement → 47.92~ 48kg nodules per kg alloy (determined by Co demand).

CO_2 from mining = 48 kg nodules × 0.00969 kg CO_2/kg nodules = 0.465 kg CO_2

The HPSR replaces carbon with hydrogen and hence in the primary extraction the direct CO_2 emissions are zero. (assuming the electricity and hydrogen are supplied from renewable sources).

Therefore, CO_2 from primary extraction = 0 kg CO_2

Total CO_2 Emissions for HPSR:

Total CO_2 = 0.465 + 0 = 0.465 kg CO_2/kg alloy

Therefore, reduction in emissions = $\frac{4.84 - 0.465}{4.84} \times 100$ = **90.39%**

If hydrogen is sourced from other pathways, such as grey or blue hydrogen, the CO_2 emissions from HPSR will increase accordingly. To estimate these emissions, we used average CO_2 intensities for each hydrogen source: 10 kg CO_2 per kg H_2 for grey hydrogen and 2.3 kg CO_2 per kg H_2 for blue hydrogen, as reported in refs (*54*, *55*). Total CO_2 emissions were then calculated by multiplying the total hydrogen consumed in the process by the respective CO_2 intensity of each hydrogen source. The results are presented in Table S4.

The collection of nodules for the HPSR and TMC NORI-D processes exhibit footprints of 0.46 and 0.40 ton CO_{2eq} per ton of alloy, respectively. These variations reflect differences in nodule composition and metal recovery efficiencies for the both processes. For instance, TMC NORI-D processes nodules which contain 1.39 wt.% Ni and attains a 95% recovery rate for Ni, whereas for the current study, nodules contain 1.2wt.% Ni and the process recovers 88% of the available Ni. Consequently, a slightly greater mass of nodules is required in the HPSR process (this study) to produce an equivalent amount of alloy. Specifically, generating 1 kg of alloy with similar composition necessitates 42 kg of nodules via TMC NORI-D and 48 kg via HPSR, as explained above.

Fig. S1.

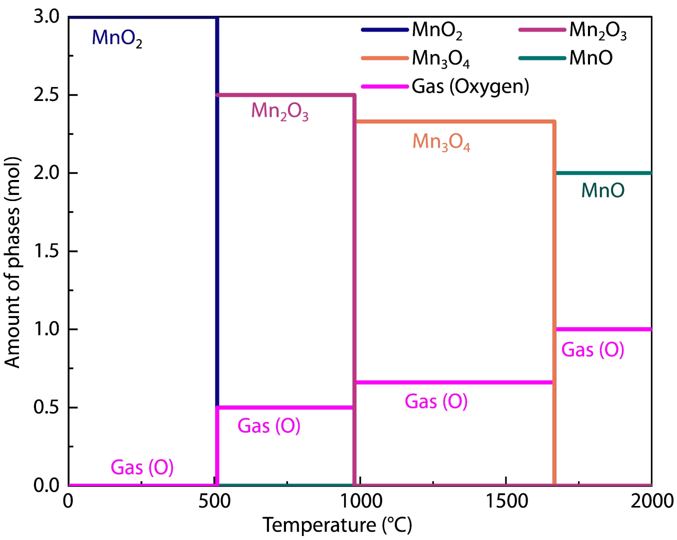

Fig. S1: Equilibrium Phase Diagram of MnO_2 Thermal Decomposition. Calculated using the Thermocalc software package 2024A in conjunction with the TCOX10 thermodynamic database, this diagram delineates the temperature-dependent phase stability of manganese dioxide (MnO_2) at 1 bar. As the system is heated, MnO_2 undergoes progressive thermal decomposition, transitioning successively to Mn_3O_4, then Mn_2O_3, and ultimately to MnO, with oxygen evolving as a gas phase in equilibrium with the condensed phases.

Fig. S2.

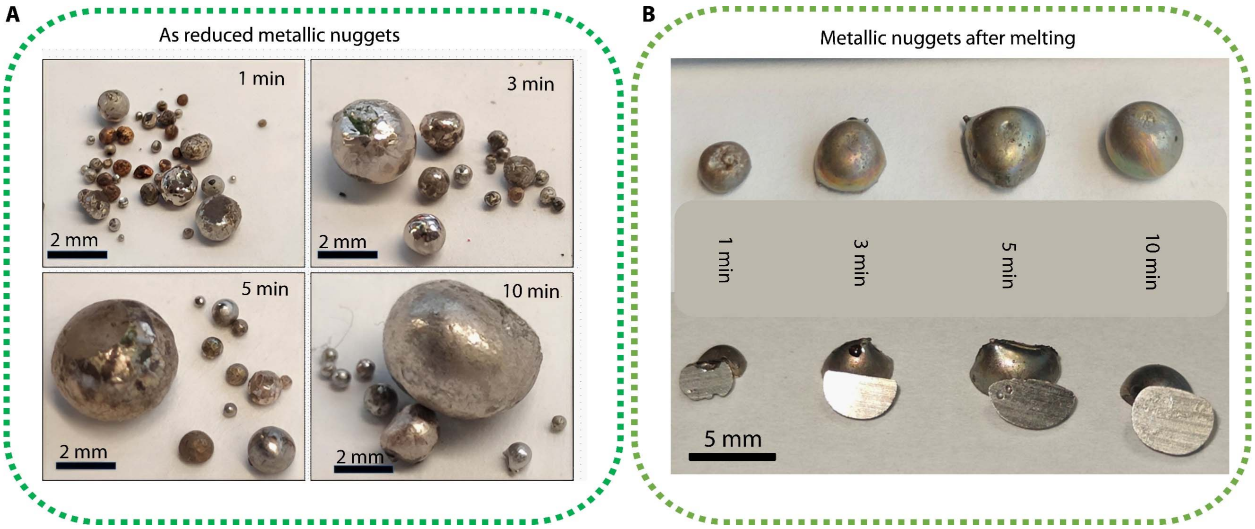

Fig. S2: Evolution and consolidation of metallic nuggets during HPSR processing. (A) Photographs of metallic nuggets formed at different processing times during HPSR treatment. **(B)** To obtain a single homogeneous metal nugget, individual nuggets from the same experiment were collected and re-melted in an inert atmosphere. The consolidated samples were subsequently sectioned for further analysis.

Fig. S3.

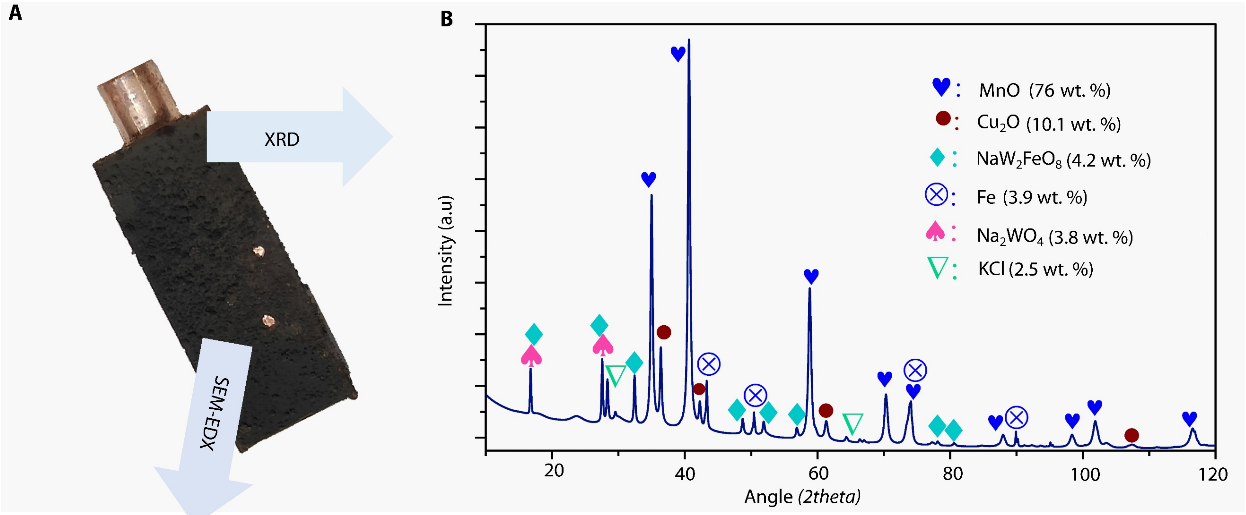

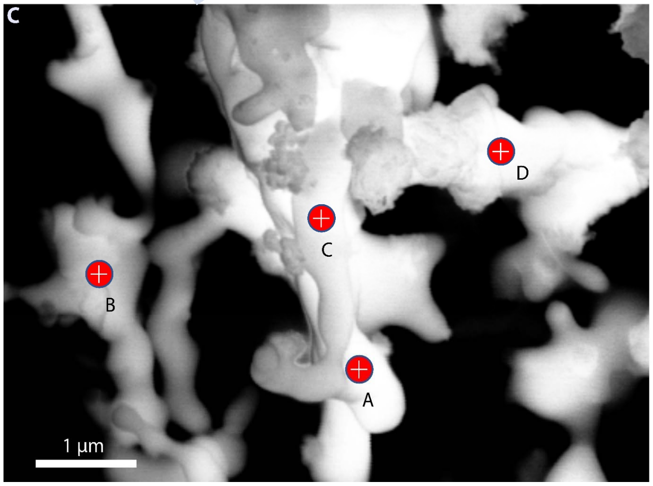

D

Element	Spot (wt.%)			
	A	B	C	D
O	17	15	24	17
Mn	73	75	65	73
Fe	4	4	4	4

Fig. S3: Characterization of vapor deposits collected from the electrostatic filter/cold finger. (A) Photograph of the electrostatic filter/cold finger showing vapor deposition on its surface. **(B)** XRD analysis of the collected deposit reveals that it is predominantly composed of the MnO phase (76 wt.%), depicted by blue heart symbol. **(C)** SEM micrograph of the vapor deposit/dust. **(D)** Corresponding point composition, as determined by EDX analysis, confirms the enrichment of the dust with Mn, and the stoichiometric ratio further validates the presence of MnO.

Fig. S4.

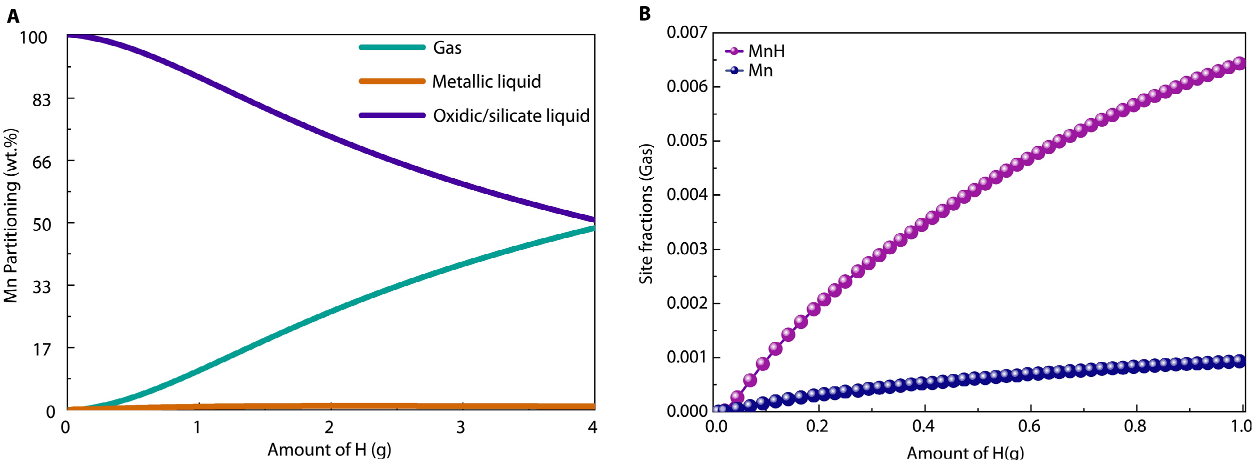

Fig. S4: Equilibrium partitioning of Mn across different phases during hydrogen exposure. (A) Evolution of Mn partitioning as Mn-nodules are exposed to increasing amounts of hydrogen. Initially, Mn exists entirely as MnO within the oxidic/silicate phase. Upon hydrogen exposure, Mn progressively transitions into the metallic liquid phase, saturating at ~2 wt.% Mn, while simultaneously partitioning into the gaseous phase. **(B)** Mn in the gas phase exists as Mn and MnH species. With increasing hydrogen exposure, the concentration of MnH also increases, lowering the partial pressure of Mn vapor and thereby enhancing Mn evaporation from the melt.

Fig. S5.

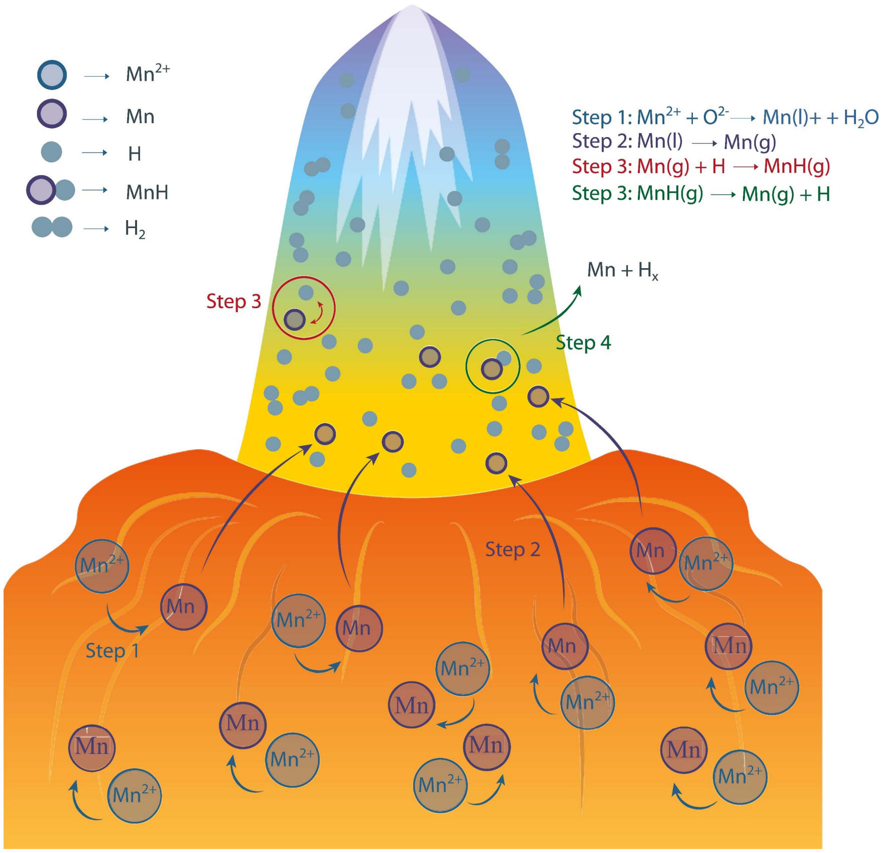

Fig. S5: **Schematic representation of Mn evaporation during hydrogen plasma smelting reduction.** For simplification purposes, only Mn species are shown inside the melt: (Step 1) MnO is reduced to metallic Mn. (Step 2) Mn evaporates into the gaseous phase due to high partial pressures at elevated temperatures (>2000°C) near the arc-melt interface. (Step 3) Mn reacts with hydrogen species in the gas phase to form MnH_x, which lowers the partial pressure of Mn vapor and enhances further Mn evaporation from the melt. Upon exiting the arc region, MnH_x dissociates into Mn and H_x, with Mn subsequently oxidizing to MnO in the presence of water vapor generated during the process.

Table S1.

Table S1: Elemental composition of as-received Mn-Nodules used for validation experiments: As-received Mn-Nodules elemental composition as determined by ICP-OES (inductive coupled plasma-optical emission spectroscopy).

**Element**	**Si**	**Mn**	**Cr**	**Ti**	**Mg**	**Fe**	**Ni**	**Al**	**Ca**	**Co**	**Cu**	**Zn**	**S**	**Na**
**Content (wt.%)**	5.5	27.5	0.1	0.4	1.7	6.6	1.2	2.1	1.2	0.12	1.0	0.3	0.02	1.5

Table S2.

Table S2: Phase composition: Phase constitution of the as-received deep-sea polymetallic nodules as determined by XRD.

Chemical formula	Mineral	Content (wt. %)
SiO_2	Quartz	3.9
$MnO_2.(0.3H_2O)$	Manganese oxide hydrate	79.4
$Na_{0.6}Ca_{0.4}Al_{1.4}Si_{2.6}O_8$	Albite	9.0
$(Mg, Fe)Al_2SiO_5(OH)_2$	Magnesiochloritoid	7.7

Table S3.

Table S3. Energy Comparisons: Input energy distribution in rotary dryers and rotary kilns (*53*).

Processing Step	Energy input (% of total energy consumed by whole process)	Energy utilized to run the process	Losses
**(a) Rotary Dryer**	15.2	10	5.2
**(b) Rotary Kiln**	42.42	24.2	18.2
**Total (a) + (b)**	57.6	34.2	23.4

Table S4.

Table S4. CO_2 emissions distribution: CO_2 emissions from different steps involved in Mn-nodules processing through RK-EF based TMC NORI-D route.(29)), and via HPSR. Primary extraction involves steps after mining like drying in rotary dryers, calcination in rotary kilns and smelting in electric arc furnaces. The electricity is assumed to be from renewable sources for both TMC NORI-D and HPSR.

Processing Step	TMC NORI-D (ton CO_{2eq}/ton alloy)	HPSR (ton CO_{2eq}/ton alloy)		
		Green H_2	Blue H_2	Grey H_2
**Collection of nodules**	0.40	0.46	0.46	0.46
**Primary extraction**	4.43	0	0.45	1.28
**Total**	4.84	0.46	0.91	1.74
